# Krill Oil Combined with *Bifidobacterium* *animalis* subsp. *lactis* F1-7 Alleviates the Atherosclerosis of ApoE^−/−^ Mice

**DOI:** 10.3390/foods10102374

**Published:** 2021-10-06

**Authors:** Xi Liang, Zhe Zhang, Youyou Lv, Haiyan Lu, Tongjie Liu, Huaxi Yi, Maozhen Zhao, Lanwei Zhang, Pimin Gong

**Affiliations:** College of Food Science and Engineering, Ocean University of China, Qingdao 266003, China; liangxi6029@163.com (X.L.); zhangzhe_hmu@126.com (Z.Z.); youyoulv666@163.com (Y.L.); luhaiyanouc@163.com (H.L.); ltjpeak@126.com (T.L.); yihx@ouc.edu.cn (H.Y.); 18846194116@163.com (M.Z.)

**Keywords:** krill oil, *Bifidobacterium animalis* subsp. *lactis* F1-7, atherosclerosis, apoE^−/−^ mice, lipid-lowering, anti-inflammatory

## Abstract

There has been an increasing number of studies on the interaction between active substances and probiotics to improve disease. Both krill oil (KO) and probiotics have the effect of improving atherosclerotic cardiovascular disease, but the combined effect has not been explored. Therefore, the purpose of this study was to explore the improvement effect of KO combined with probiotics on atherosclerosis. The atherosclerotic plaque area of ApoE^−/−^ mice was detected after the intervention of KO, *Bifidobacterium animalis* subsp. *lactis* F1-7 (*Bif. animalis* F1-7), and KO combined with *Bif. animalis* F1-7. The results showed that *Bif. animalis* F1-7, KO, and KO combined with *Bif. animalis* F1-7 could significantly reduce the area of atherosclerotic plaque and improve the levels of serum lipids and inflammatory factors. They could regulate the farnesoid X receptor (FXR)/cholesterol 7-alpha hydroxylase (CYP7A1) pathway to reduce lipid accumulation. The intervention groups could also improve the inflammatory response by downregulating the Toll-like receptor 4 (TLR4)/myeloid differentiation factor 88 (MyD88) pathway. The anti-inflammatory effect of the interaction group was significantly better than that of KO. It proved that *Bif. animalis* F1-7 might play a synergistic effect in the improvement of inflammation by KO to the alleviation of atherosclerosis.

## 1. Introduction

Atherosclerosis is a pathological change characterized by the accumulation of blood lipids in the arteries [1], accompanied by a chronic inflammatory response [2]. In recent years, it has been found that the structural changes of intestinal flora affect the occurrence and development of atherosclerosis [3]. The metabolites of bacterial flora, such as the short-chain fatty acids, secondary bile acid, and choline metabolite TMAO, participate in regulating the inflammatory response and lipid metabolism, thus aggravating or improving the disease state [4,5].

Krill oil (KO) extracted from Euphausia superba has many health-promoting properties, such as anti-inflammatory, hypoglycemic, and lipidemic effects [6,7]. Krill oil proved to be effective in alleviating atherosclerosis in apoE^−/−^ mice [8]. KO contains docosahexaenoic acid (DHA) and eicosapentaenoic acid (EPA), which are important components of omega-3 polyunsaturated fatty acids and have a variety of biological effects, including lipid-lowering and antithrombotic effects [9,10]. KO could affect the different flora of the gastrointestinal tract and increase the abundance of *Lactobacillus*, so it alleviated the gut microbiota dysbiosis and increased the levels of short-chain fatty acid production, as well as improved diseases such as hyperlipidemia [11]. In addition, probiotics could also regulate the gut flora to ameliorate the lipid metabolism and inflammatory response caused by atherosclerosis [12]. We have previously found a number of probiotics with lipid-lowering functions such as *Bifidobacterium animalis* subsp. *lactis* F1-7 (*Bif. animalis* F1-7) [13]. The probiotics with BSH activity could regulate secondary bile acid products, which act on the key targets of bile acid metabolism and improve the lipid level [14]. 

However, the effect of KO combined with probiotics on atherosclerosis in vivo has not yet been investigated, which has certain research significance and commercial value. Therefore, the purpose of this study was to investigate the interaction effect between the active substance KO and functional probiotic *Bif. animalis* F1-7. We explored the mechanism of KO combined with *Bif. animalis* F1-7 on the lipid metabolism and inflammatory changes for alleviating atherosclerosis. This study provides a new research direction for the prevention and treatment of atherosclerotic-related cardiovascular diseases by nontraditional methods.

## 2. Materials and Methods

### 2.1. KO Samples and Composition Analysis

KO samples were provided by Shandong Luhua Marine Biotechnology Co., Ltd. (Jinan, China). The fatty acid composition of krill oil was measured via gas chromatography [15,16]. The KO samples were stored at 4 °C. Before use, the appropriate amount of KO was weighed and diluted with PBS or PBS containing probiotics.

### 2.2. Strains and Culture

The experimental strain *Bif. animalis* F1-7 (NO. CCTCC M 2020833) was stored in our laboratory. The *Bif. animalis* F1-7 was incubated and activated twice in MRS at 37 °C, and then centrifugated at 5000× *g* for 15 min. The *Bif. animalis* F1-7 was washed twice with PBS, the colony count method was used to adjust the total viable count to 1 × 10^8^ CFU/mL, and the suspension was stored at 4 °C for standby.

### 2.3. Animal and Experimental Design

All experiments were performed in accordance with the British Animal (Scientific Procedures) Act 1986 (PPL 70/7652) and were approved by the Laboratory Animal Ethics Committee of the Ocean University of China (approval number: SPXY2020060502). A number of 24 male AopE^−/−^ mice on a C57BL background at 8 weeks of age were purchased from Beijing Vital River Laboratory Animal Technology Co., LTD (Beijing, China). After being adaptively fed for 7 days, the AopE^−/−^ mice were randomly divided into 4 groups with 6 mice in each group, and the specific groups were as follows: (1) atherosclerosis group (AS): 0.25 mL of PBS gavage daily; (2) *Bif. animalis* F1-7 intervention group (F1-7): 0.25 mL of daily gavage containing 1 × 10^8^ CFU probiotic; (3) KO group (KO): 0.25 mL of KO solvent (1.5 mg KO/day); (4) KO + *Bif. animalis* F1-7 intervention group (KF): 0.25 mL of daily gavage containing 1.5 mg of KO and 1 × 10^8^ CFU probiotic agents. The high-fat diet was provided by Beijing Keao Xieli Feed Co., LTD (Beijing, China), and the composition of high-fat feed is shown in Table 1. The mice were exposed to a 12 h light/dark cycle, and was maintained at a temperature of 23 ± 2 °C and humidity of 65 ± 15%. Mice were fed a high-fat diet during the experiment and were treated with the *Bif. animalis* F1-7, KO, and KO combined with *Bif. animalis* F1-7 from week 6. The experiment lasted for 16 weeks. The day before the end of the experiment, adequate feces of mice were collected and stored aseptically at −80 °C. The mice fasted overnight. After the mice were anesthetized, the plasma was collected and centrifuged at 4000× *g* for 15 min. The upper serum was stored at −80 °C before use. The liver and intestinal tissues were collected and part of the tissues were fixed with 4% paraformaldehyde fixative, the other part was preserved for other determination. The aortas, aortic roots, and arches were excised for histological examination.

### 2.4. Aortic Oil Red O Staining

The aorta (including aortic arch and thoracic and abdominal regions) was opened longitudinally and fixed with 4% paraformaldehyde overnight at 4 °C. Then, it was stained according to the oil red O staining procedure [17]. Images of the aorta were taken with a digital camera and the aortic plaque area was analyzed with Image J software.

### 2.5. Histological and Immunohistochemical Analysis

After the aortic and liver tissue were fixed with 4% paraformaldehyde fixative, it was embedded and then sliced into 4 μm sections. The paraffin-embedded sections were deparaffinized and rehydrated, and they were then spread for H&E staining according to the procedure [18]. The slices were sealed with neutral resin and photographed using a microscope (Olympus, Center Valley, PA, USA). Immunohistochemical analysis [19] was performed to analyze the expression levels of F4/80, NF-κB, TLR4, and MyD88 of the aorta (1:100, Absin, Zhang Jiang Hi-Tech Park, Pudong, Shanghai, China). The histological assessments were carried out by an independent researcher.

### 2.6. Biochemical Analysis

The serum total cholesterol (TC), triglyceride (TG), high-density lipoprotein (HDL), and low-density lipoprotein (LDL) levels were measured using corresponding kits (Jiancheng, Nanjing, China). Inflammatory mediator lipopolysaccharide (LPS) and inflammatory molecules including TNF-α and IL-1β levels in serum were determined with the ELISA kit (Jiancheng, Nanjing, China) according to the instructions.

### 2.7. Determination of Total Bile Acids in Feces

After freeze-drying with the vacuum freezer (Christ, Frankfurt, Germany), a 0.5 g sample of feces was taken, mixed with 5 mL of deionized water, and then centrifuged at 4000× *g* for 10 min. The supernatant was taken and measured with the total bile acid (TBA) assay kit (Jiancheng, Nanjing, China) [20].

### 2.8. Gene Expression Analysis

Total RNA was extracted from the liver or ileum with TRIzol reagent (Invitrogen, Carlsbad, CA, USA), followed by reverse transcription into cDNA with the reverse transcription kit (Invitrogen, Carlsbad, CA, USA). Quantitative real-time polymerase chain reaction was performed with a PCR reverse transcription instrument (Life Technologies, Paisley, UK). The primers for each specific gene are listed in Table 2.

### 2.9. Statistical Analysis

All analyses were performed on three parallel samples, and the data for each variable were expressed as mean ± standard deviation. The data were evaluated by one-way analysis of variance (ANOVA) and Duncan analysis. *p* < 0.05 was considered a statistically significant difference.

## 3. Results

### 3.1. The Compositions Analysis of Krill Oil

The physicochemical properties and fatty acids of KO were analyzed and are summarized in Table 3. The KO had high contents of phospholipids (63.54%). It contained a large amount of polyunsaturated fatty acids (PUFA), especially DHA and EPA, which were 29.36% and 19.02%, respectively. In addition, the KO with high astaxanthin (190.25 mg/kg) had better anti-inflammatory and anti-oxidant effects. 

### 3.2. The KO Combined with Bif. animalis F1-7 Reduced the Formation of Atherosclerotic Lesion in Apoe^−/−^ Mice

**Figure 1:**
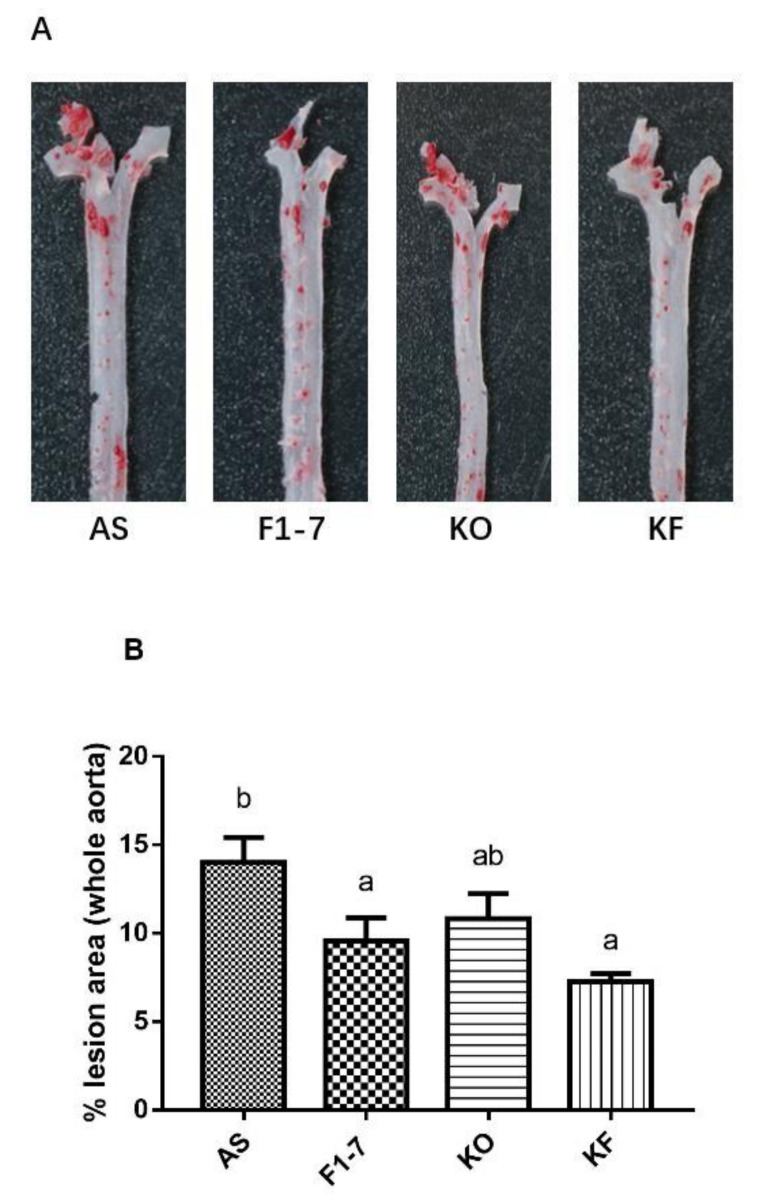

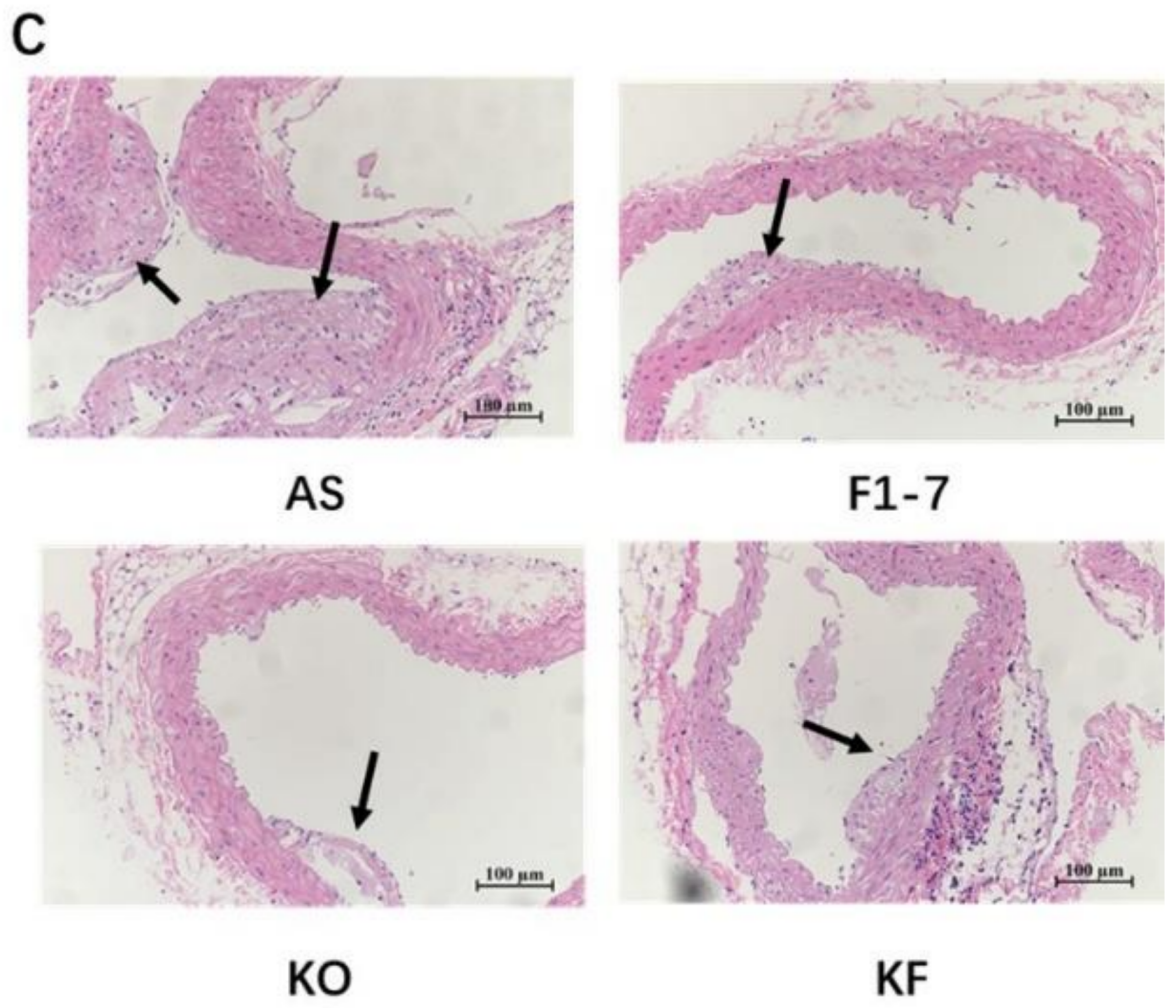
(**A**) Representative images of the aorta Oil Red O staining plaque burden (n = 4). (**B**) The quantification of lesion in percentage area was analyzed by Image J software. Different letters indicate significant differences from each group, *p <* 0.05. (**C**) H&E staining of the aorta section (n = 3). The arrow points to the plaque of the aorta.

After intervention with KO and probiotics, the results of the oil red O staining of aorta are shown in Figure 1A. The atherosclerotic lesions of mice were aggravated in the AS group. The intervention of *Bif. animalis* F1-7, KO, and KO combined with *Bif. animalis* F1-7 significantly reduced the scope of the atherosclerotic lesion. The analysis of plaque coverage in Figure 1B showed that, compared with the AS group, treatment with *Bif. animalis* F1-7 reduced the lesion area by 32% and with KO by 23%. The plaque area in the KF group was reduced by 48%. H&E staining of the aorta section analysis confirmed that, in the AS group, the cardiovascular endothelial cells of the AopE^−/−^ mice were widely injured and lipid deposition was observed. The inner wall of the aorta was obviously marked with large plaques (Figure 1C). In the *Bif. animalis* F1-7, KO, and KF groups, the cardiovascular membranes of mice were slightly damaged and a small amount of plaque was attached.

### 3.3. The KO Combined with Bif. animalis F1-7 Reduced the Lipid Accumulation in Liver

**Figure 2:**
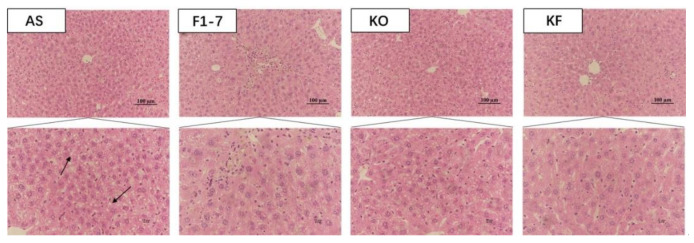
The representative photographs for H&E staining in the livers of mice (n = 4). Scale bar: 100 μm. The arrow points to the fat droplets.

H&E staining of liver tissues in mice showed that the hepatocytes of mice in the AS group were swollen, with loose cytoplasm and lipid droplet vacuoles of different sizes (Figure 2). All intervention groups were able to improve the lipid accumulation in the liver and alleviate the degree of liver steatosis. The KF group had less lipid deposition in hepatocytes, and the effect was significantly better than those of *Bif. animalis* F1-7 and KO groups. 

### 3.4. The KO Combined with Bif. animalis F1-7 Alleviated the Serum Lipid Levels in Atherosclerotic Mice

**Figure 3:**
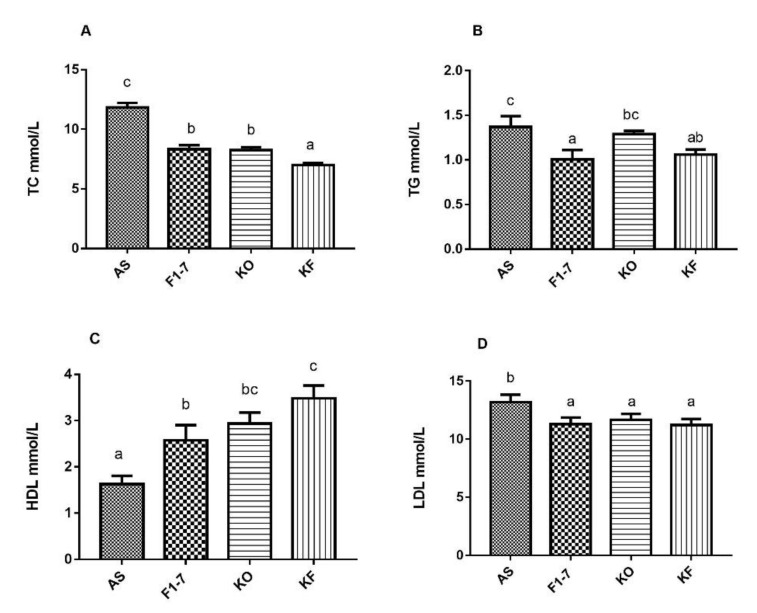
Influence of KO and the mixture of KO and *Bif. animalis* F1-7 on serum lipids (n = 6). (**A**) TC, (**B**) TG, (**C**) HDL, and (**D**) LDL. Different letters indicate significant differences from each group, *p <* 0.05.

The serum lipid metabolism in the atherosclerosis model mice was disturbed, and the cholesterol content was increased to 11.82 ± 0.95 mmol/L (Figure 3A). After the intervention of *Bif. animalis F1-7*, the TC content in serum could be reduced by 29%, no significant difference compared with KO group. The KO combined with *Bif. animalis* F1-7 intervention could reduce the TC content in serum by 40%. A high-fat diet resulted in elevated serum TG levels in Aope^−/−^ mice (Figure 3B). The *Bif. animalis* F1-7 and the KO combined with *Bif. animalis* F1-7 intervention group could effectively decrease the serum TG of atherosclerosis mice compared with the AS group. All the intervention groups significantly increased serum HDL levels of atherosclerotic mice (Figure 3C). Compared with the AS group, HDL levels were increased by 51% in the KO group. The HDL of the KF group was significantly higher than that of the *Bif. animalis* F1-7 group. The KF group increased the HDL by 63% compared with the AS group. In addition, the mixture of KO and *Bif. animalis* F1-7 could effectively downregulate LDL levels in serum of the Apoe^−/−^ mice (Figure 3D). The effect of the *Bif. animalis* F1-7 group and KO group were not significant compared with the AS group.

### 3.5. Effects of the KO Combined with Bif. animalis F1-7 on the Total Bile Acids Level in Feces

**Figure 4:**
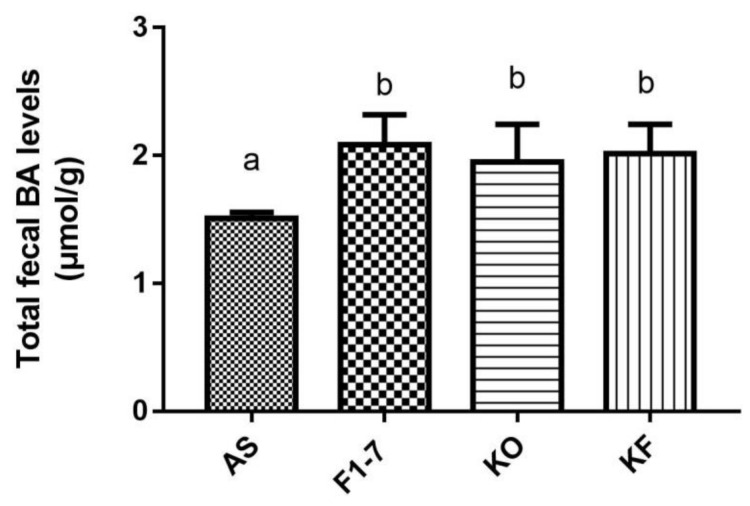
Total bile acid (TBA) levels of mice in feces (n = 4). Different letters indicate significance (*p* < 0.05).

After the intervention of *Bif. animalis* F1-7, KO, and KO combined with *Bif. animalis* F1-7, the total bile acid content in the feces of atherosclerotic mice was significantly increased (Figure 4). There was no statistical difference between the *Bif. animalis* F1-7, KF, and KO groups. The mixture of KO and *Bif. animalis* F1-7 effectively promoted bile acid metabolism, increased bile acid efflux in mice, and thus participated in the regulation of lipid level in mice. However, the effect of the KO combined with *Bif. animalis* F1-7 was not superior to the effect of KO or *Bif. animalis* F1-7.

### 3.6. Effects of KO Combined with Bif. animalis F1-7 on the Expression of Key Genes in Lipid Metabolism

**Figure 5:**
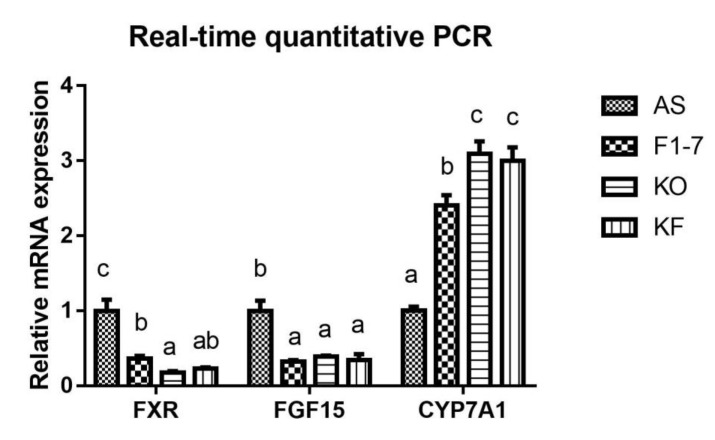
Relative mRNA levels of intestine FXR, FGF15, and liver CYP7A1 genes involved in lipid metabolism (n = 4). Different letters indicate significant differences from each group, *p <* 0.05.

The expression of key genes of lipid metabolism in atherosclerotic mice was determined (Figure 5). Compared with the AS group, the *Bif. animalis* F1-7 and KO interventions were found to effectively downregulate the expression of FXR in the intestinal tract of Apoe^−/−^ mice (*p* < 0.05). The KO combined with *Bif. animalis* F1-7 intervention could downregulate the expression of intestinal FXR, but there was no significant difference from the other two groups. In addition, the *Bif. animalis* F1-7, KO, and KF groups downregulated the expression of FGF15 in the intestinal tract of atherosclerosis mice induced by high-fat diet and could effectively improve the expression of CYP7A1 in the liver (*p* < 0.05). 

### 3.7. The KO Combined with Bif. animalis F1-7 Reduced the Inflammatory Response in Serum of Atherosclerosis Mice

**Figure 6:**
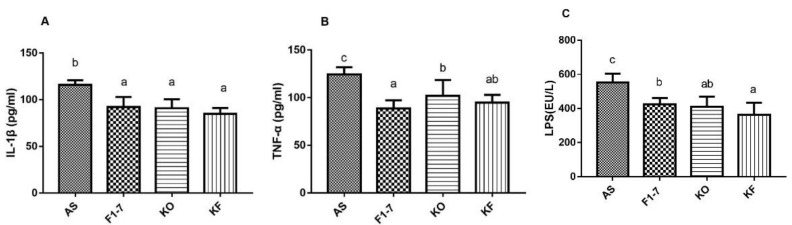
The plasma IL-1β (**A**), TNF-α (**B**), and LPS (**C**) levels were measured by ELISA (n = 5). There were statistically significant differences between groups of different letters, *p* < 0.05.

The level of inflammatory changes in atherosclerotic mice was measured, and KO and the KO combined with *Bif. animalis* F1-7 were found to effectively decrease the expression of serum inflammatory factors in mice (Figure 6). The serum level of IL-1β and was significantly reduced in the *Bif. animalis* F1-7, KO, and KF groups compared with the AS group. There was no statistical difference between the three interventions. *Bif. animalis* F1-7 could effectively improve the serum TNF-α level of ApoE^−/−^ mice, and its effect was better than that of the KO group. Compared with the AS group, serum TNF-α in the KF group was downregulated by 24%, and the effect was not statistically different from *Bif. animalis* F1-7. The LPS levels in the serum of the *Bif. animalis* F1-7, KO, and KF group were decreased compared with the AS group (*p* < 0.05). The effect of KO combined with *Bif. animalis* F1-7 was better than that of the *Bif. animalis* F1-7 group. The KO and *Bif. animalis* F1-7 groups showed no statistical difference.

### 3.8. Effects of KO Combined with Bif. animalis F1-7 on the Targets Associated with Inflammatory Responses to Aortic Sinus

**Figure 7:**
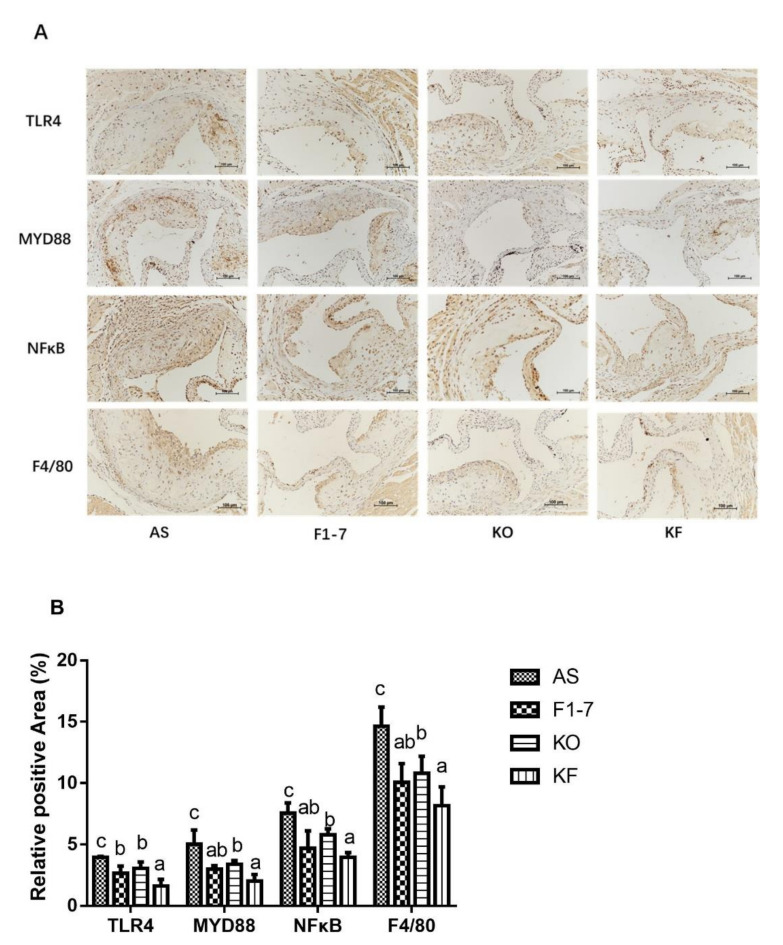
(**A**) TLR4, MYD88, NFκB, and F4/80 immunohistochemistry staining of the aortic sinus in atherosclerotic mice (n = 4), and (**B**) quantitative analysis of TLR4, MYD88, NFκB, and F4/80. Scale bar, 100 μm. Different letters indicate significant differences, *p* < 0.05.

Immunohistochemical staining of TLR4 showed that in the atherosclerosis model group, the TLR4-positive regions in the *Bif. animalis* F1-7, KO, and KF groups were significantly reduced (Figure 7). Compared with the AS group, the KO group decreased the expression of TLR4 by 22%, with no statistical difference from the *Bif. animalis* F1-7 group. The KF group decreased by 59%, better than that of the *Bif. animalis* F1-7 and KO groups (*p* < 0.05). Both intervention groups could decrease the expression of MYD88 and reduce the pro-inflammatory signal transduction, and the KF group had a better effect than the KO group. There was no significant difference between KF and *Bif. animalis* F1-7 groups. The results of NFκB showed that the intervention groups could improve the inflammatory expression of atherosclerosis mice. *Bif. animalis* F1-7 intervention decreased the NFκB by 38%. After KO intervention, the NFκB expression was decreased by 24% compared with the AS group. The KO combined with *Bif. animalis* F1-7 intervention downregulated the NFκB expression by 47%, and the KF group was better than that of the KO group. The interventions of *Bif. animalis* F1-7, KO, and KO combined with *Bif. animalis* F1-7 could effectively reduce the positive area of F4/80 in the aortic sinus of atherosclerotic mice, and improve the accumulation of macrophages and inflammatory response. The effect of KO combined with *Bif. animalis* F1-7 was better than that of KO.

### 3.9. KO Combined with Bif. animalis F1-7 Decreased the Gene Expression of Key Factors Regulating Inflammatory Response in the Intestine of Atherosclerosis Mice

**Figure 8:**
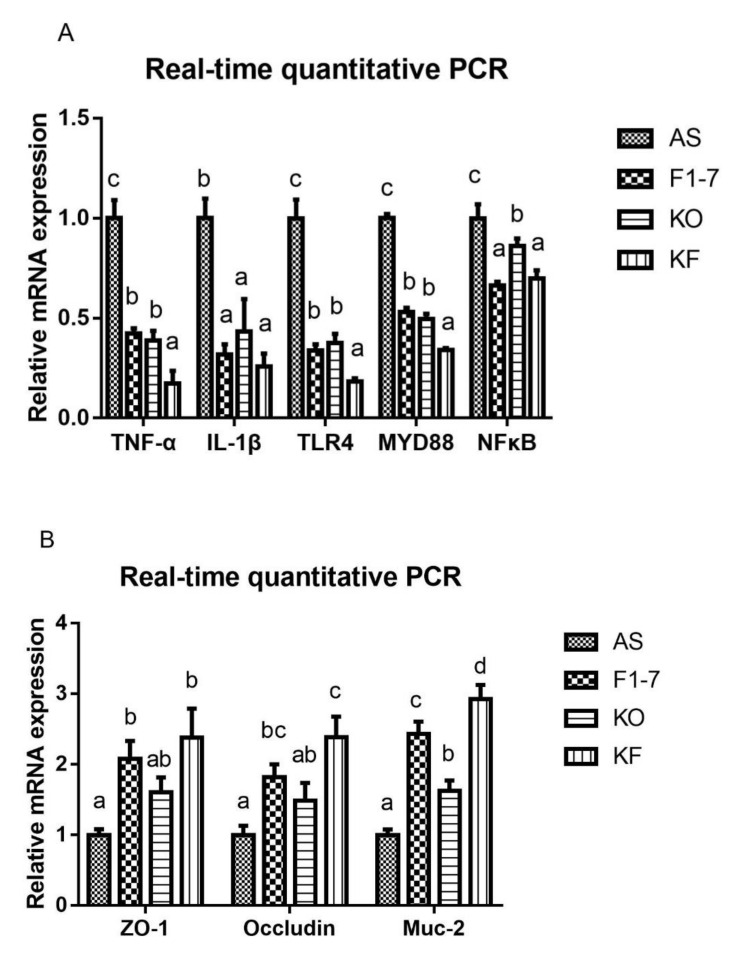
(**A**) Relative mRNA levels of the inflammatory factors in the intestine, containing the genes of TNF-α, IL-1β, TLR4, MYD88, and NFκB, and (**B**) mRNA expression of epithelial integrity proteins, Muc-2, occludin, and zonula occludens-1 (ZO-1) (n = 3). Different letters indicate significant differences, *p* < 0.05.

The expression of genes related to inflammatory response was determined (Figure 8A). Compared with the AS group, the expression of TNF-α was decreased in the *Bif. animalis* F1-7 group and KO group. The effect of the KF group was significantly better than that of the *Bif. animalis* F1-7 and KO groups. IL-1β expression was reduced after the intervention of *Bif. animalis* F1-7, KO, and the KO combined with *Bif. animalis* F1-7. There was no significant difference among groups. The intervention groups could regulate the expression of key genes in the TLR4/MYD88/NF-κB pathway, and the downregulation effect of KF was significantly superior to that of the *Bif. animalis* F1-7 or KO group on regulating TLR4 and MYD88. The KF group decreased the expression of NFκB, which had a better effect than the KO group, and there was no significant difference with the *Bif. animalis* F1-7 group. The integrity of the intestinal epithelial cell barrier was analyzed (Figure 8B). Compared with the AS group, the zonula occludens-1 (ZO-1) mRNA level in *Bif. animalis* F1-7 and KO combined with the *Bif. animalis* F1-7 group was significantly increased (*p* < 0.05). There was a trend toward an increase in ZO-1 mRNA expression by KO. However, there was no statistical difference with the model group. The mRNA levels of occludin were upregulated in three intervention groups, and the effect of KO combined with the *Bif. animalis* F1-7 group was most significant compared with the AS group. KO and *Bif. animalis* F1-7 could increase the mRNA level of Mucin-2 (Muc-2) (*p* < 0.05). The effect of KO combined with *Bif. animalis* F1-7 was significantly better than those of the other two intervention groups (*p* < 0.05).

## 4. Discussion

Atherosclerosis accompanied by lipid metabolism disorder [21] and chronic systemic inflammatory infiltration [22] can lead to serious cardiovascular diseases and related complications. Inflammation causes vascular endothelial dysfunction and monocytes/macrophages to infiltrate the inner walls of the vessels, accelerating AS [23]. This study showed that the *Bif. animalis* F1-7, KO, and KO combined with *Bif. animalis* F1-7 interventions significantly reduced the scope of atherosclerosis.

KO is widely believed to have the effects of lowering the lipids level, increasing anti-inflammatory effects, and improving cardiovascular diseases [8]. KO contains large amounts of phospholipids that had a better effect on the bioavailability of EPA and DHA [24]. It has been reported that its main components EPA and DHA play key roles in metabolic regulation [25]. In addition, KO also contains a dose of astaxanthin, which is believed to be responsible for the anti-inflammatory, antioxidant, analgesic, and lipid-lowering properties in vivo [26,27]. It has been found that KO improves intestinal flora structure and metabolites. For example, KO inhibits key metabolites of histidine metabolism of the host and microorganism, contributing to its anti-inflammatory activity [28]. KO reduces inflammatory factors such as TNF-α and IL-1β by regulating the NF-κB pathway. Lipopolysaccharide (LPS) participated in various intestinal epithelial signaling pathways through TLRS and promoted the production of various inflammatory factors. TLR4 binds to LPS to form the TLR-4/CD14/LBP receptor complex. By acting on the MYD88 receptor, the complex activates NF-κB expression and promotes the expression of downstream inflammatory factors such as IL-1β and TNF-α [29]. In addition, the inflammation was associated with impaired mucosal epithelial integrity and barrier function. The mucins Mucin-2 and Mucin-3, as well as occludin and ZO-1, are systematically involved in various enteral and parenteral diseases via endotoxemia regulation [30]. Proinflammatory cytokines produced by macrophages act directly on intestinal epithelial cells to increase membrane permeability. KO’s ability to restore impaired barrier function may be related to its inhibition of cytokine production [31]. KO was found to effectively improve the inflammatory response in atherosclerotic mice, restore impaired barrier function, and reduce plaque accumulation by regulating the TLR4/MYD88/NF-κB pathway in this study. We found that KO combined with *Bif. animalis* F1-7 had a better effect on reducing the inflammatory response than KO did, where *Bif. animalis* F1-7 might play an important role. Previous studies have shown that the probiotics and their metabolites had the abilities to improve the inflammatory response and intestinal barrier [32,33,34,35], and reduce atherosclerotic plate accumulation [36,37]. Therefore, we hypothesized that KO interacts with probiotics to produce some specific changes that affect the involvement of intestinal flora or metabolites in anti-inflammatory effects. 

The FXR is a key target of bile acid metabolism and is involved in lipid metabolism regulation. Intestinal FXR regulates liver CYP7A1 by acting on the fibroblast growth factor 15 (FGF15) and improves bile acid metabolism [38,39]. Our previous study found that probiotic *Bif. animalis* F1-7 could downregulate intestinal FXR, thus promoting bile acid metabolism [40]. KO could participate in lipid metabolism by regulating key targets of lipid metabolism and improving intestinal flora [41,42]. In this study, it was found that KO and KO combined with *Bif. animalis* F1-7 downregulated the expression of intestinal FXR and FGF15, and in response, regulated the expression of CYP7A1 in the liver. They promoted the excretion of excessive bile acids through feces and increased the metabolism of bile acid, thus reducing the cholesterol level and improving lipid metabolism. However, in this study, the lipid-lowering effect of the KO combined with *Bif. animalis* F1-7 group was not significantly better than that of the *Bif. animalis* F1-7 or KO group. These results indicated that, although KO and *Bif. animalis* F1-7 both had lipid-lowering effects, they could not exert superimposed effects on the FXR/CYP7A1 signaling pathway. In addition, combined with anti-inflammatory effects, we speculated that the mixture of KO and *Bif. animalis* F1-7 effectively regulated inflammatory responses and reduced the accumulation of aortic plaque in ApoE^−/−^ mice by producing some anti-inflammatory substances. 

In previous studies, we found that *Bif. animalis* F1-7 had high hydrophobicity and self-aggregation [43] and had ability to colonize in the intestine through fluorescence colonization experiments [44]. This suggests that F1-7 has a direct probiotic effect in the gut. Probiotics can improve the structure of bacteria and affect the composition and content of metabolites [45]. Through full-spectrum metabonomics, we found that *Bif. animalis* F1-7 had significant effects on carnitine metabolism. In addition, its anti-inflammatory effect was mainly regulated by the derivative of carnitine. In addition, the results of the current study suggest that *Bif. animalis* F1-7 does not exert its anti-inflammatory effects by producing short-chain fatty acids. We speculated that this finding may be related to the effect of *Bif. animalis* F1-7’s synergistic KO on the inflammatory response in this study. However, the regulation of intestinal flora is a delicate and complex process. Further analysis is still needed to determine whether this particular metabolite is produced by the decomposition of KO by the strain or is caused by the structural change in the flora. Their specific mechanisms need to be further studied in combination with metabolomics and intestinal flora analysis.

## 5. Conclusions

KO combined with *Bif. animalis* F1-7 effectively improved the lipid metabolism disorder and inflammatory response associated with atherosclerosis. It could effectively improve the inflammatory response by acting on the TLR4/MYD88/NF-κB pathway. It also promoted bile acid metabolism and regulated lipid levels through the FXR/FGF15/CYP7A1 pathway, ultimately improving atherosclerosis. Compared with *Bif. animalis* F1-7 and KO, KO combined with *Bif. animalis* F1-7 showed obvious advantages in anti-inflammatory effects. The *Bif. animalis* F1-7 further improved the ability of KO to protect the intestinal mucosal barrier and reduce inflammation, thereby improving AS. The study provided a new research direction for the prevention and treatment of atherosclerotic vascular diseases. However, the interaction mechanism and specific targets of KO and probiotics still need to be further explored and clarified.

## Figures and Tables

**Table 1:** Composition of normal and high-fat diets.

	Normal Diet	High Fat Diet
Nutrient and energy composition	Caloric (kcal/100 g) 352	Caloric (kcal/100 g) 524
	Carbohydrates (g/100 g) 52	Carbohydrates (g/100 g) 20
	Energy (%) 63.9	Energy (%) 20
	Proteins (g/100 g) 20.1	Proteins (g/100 g) 28
	Energy (%) 20.3	Energy (%) 35
	Lipids (g/100 g) 5.9	Lipids (g/100 g) 33
	Energy (%) 15.8	Energy (%) 45
(g/kg)	Casein 200	Casein 200
	l-Cystine 3	l-Cystine 3
	Corn starch 397	Corn starch 72.8
	Maltodextrin 132	Maltodextrin 100
	Sucrose 100	Sucrose 172.8
	Cellulose 50	Cellulose 50
	Soybean Oil 70	Soybean Oil 25
	*t*-Butylhydroquinone 0.014	*t*-Butylhydroquinone 25
	Lard Oil 0	Lard Oil 177.50
	Mineral Mix 35	Mineral Mix 10
	Vitamin Mix 10	Vitamin Mix 10
	Choline Bitartrate 2.5	Choline Bitartrate 2
	Potassium Bitartrate 0	Potassium Bitartrate 16.50
	Calcium Carbonate 0	Calcium Carbonate 5.5
	FD&C Red Dye 0	FD&C Red Dye 0.05
	Di Calcium Phosphate 0	Di Calcium Phosphate 13

**Table 2:** RT-PCR amplified primers.

Gene Name	Forward (5′-3′)	Reverse (5′-3′)
β-actin	ACTGCTCTGGCTCCTAGCAC	CCACCGATCCACACAGAGTA
FXR	ACAGAGAGGCGGTGGAGAAGC	TCAGCGTGGTGATGGTTGAATGTC
FGF15	CCTGTACTCCGCTGGTCCCTATG	GGTCCTCCTCGCAGTCCACAG
TLR4	CACAGAAGAGGCAAGGCGACAG	GAATGACCCTGACTGGCACTAACC
MYD88	AGCAGAACCAGGAGTCCGAGAAG	GGGCAGTAGCAGATAAAGGCATCG
NF-κB	TCGAGTCTCCATGCAGCTACGG	CGGTGGCGATCATCTGTGTCTG
TNF-α	GGACTAGCCAGGAGGGAGAACAG	GCCAGTGAGTGAAAGGGACAGAAC
IL-1β	TCGCAGCAGCACATCAACAAGAG	AGGTCCACGGGAAAGACACAGG
Occludin	ACCCGAAGAAAGATGGATCG	CATAGTCAGATGGGGGTGGA
ZO-1	CTTCTCTTGCTGGCCCTAAAC	TGGCTTCACTTGAGGTTTCTG
Muc-2	ATGCCCACCTCCTCAAAGAC	GTAGTTTCCGTTGGAACAGTGAA
CYP7A1	GTGTAGAGGCTGGAGGTGATGTTG	AAGGGCACTGCGGCAAGTTG

**Table 3:** Physicochemical properties and fatty acid compositions of KO.

Compositions and Properties		%
Fatty acid	C12:0	0.13
	C14:0	9.06
	C16:0	12.32
	C16:1	5.96
	C17:0	0.11
	C18:0	1.63
	C18:1	10.91
	C18:2	1.92
	C18:3	1.52
	C22:0	0.09
	C20:4	1.01
	EPA C20:5	29.36
	DHA C22:6	19.02
	Phospholipids (%)	63.54
	Astaxanthin(mg/100 g)	190.25
	Acid value (mg KOH/g)	6.23
	Peroxide value (meq/kg)	2.05
